# Early and repetitive novel-tracer PET-guided stereotactic body radiotherapy for nodal oligorecurrent prostate cancer after definitive first-line therapy

**DOI:** 10.1007/s00066-024-02304-9

**Published:** 2024-09-27

**Authors:** Arne Grün, Selin Cumaoglu, Anne Kluge, Thorsten Schlomm, Dirk Böhmer, Kurt Miller, Holger Heidenreich, Daniel Zips, Goda Kalinauskaite

**Affiliations:** 1 Department for Radiation Oncology, Campus Virchow-Klinikum, Charité—Universitaetsmedizin Berlin, Corporate member of Freie Universitaet Berlin, Humboldt-Universitaet zu Berlin and Berlin Institute of Health, Augustenburger Platz 1, 13353 Berlin, Germany; 2 MVZ Leipzig Strahlentherapie, Landsberger Straße 81, 04157 Leipzig, Germany; 3 Department for Urology, Charité—Universitätsmedizin Berlin, corporate member of Freie Universitaet Berlin, Humboldt-Universitaet zu Berlin, and Berlin Institute of Health, Berlin, Germany; 4 Department for Urology, Bundeswehr Krankenhaus Berlin, Scharnhorststraße 13, 10115 Berlin, Germany

**Keywords:** Non-invasive, Metastases-directed therapy (MDT), Stereotactic ablative body radiotherapy (SABR), Postponement of hormonal therapy, Adverse events

## Abstract

**Background:**

Prostate-specific membrane antigen (PSMA) positron-emission tomography (PET) imaging can detect prostate cancer (PCa) nodal oligorecurrences (NOR) at very low prostate-specific antigen (PSA) levels. Prospective studies on oligorecurrent (OR) PCa have been hampered by either dated diagnostics or inhomogeneous cohorts and/or treatment approaches. We hypothesized that early and—if necessary and feasible—repetitive PSMA-PET-based metastasis-directed therapy (MDT) using stereotactic body radiotherapy (SBRT) would improve freedom from palliative (systemic) therapy at low toxicity.

**Methods:**

This study is a retrospective analysis of patients treated for OR PCa after definitive first-line therapy using PSMA-PET/CT-based SBRT. Endpoints were biochemical progression-free survival (bPFS), SBRT-free survival (SBRT-FS), androgen deprivation therapy (ADT)-free survival (ADT-FS), and toxicity.

**Results:**

A total of 67 patients and 248 metastases (211 nodal) were treated. Patients on concurrent ADT were excluded. Median PSA at inclusion was 2.175 ng/ml. bPFS, SBRT-FS, and ADT-FS for multiple-course SBRT were 9.5, 19.5, and 35.0 months, respectively; 32 patients had ≥ 1 course of SBRT. Median PSA nadir was 0.585 ng/ml. There was no ≥ grade 2 toxicity.

**Conclusion:**

Modern-tracer PET/CT-based early and repetitive focal SBRT yields promising results with regard to bPFS, SBRT-FS, and ADT-FS with low toxicity. The ability of this approach to postpone initiation of palliative treatment with low toxicity should be re-evaluated prospectively.

## Background

Metastatic spread is the main cause of cancer-related death [1]. It occurs rather in the form of spread between distant sites than from the primary tumor [2, 3]. Oligo-recurrences (OR) [4–6] often predate further dissemination. The responsible clone for lethal spread can originate in every metastasis [7]; hence, metastasis-directed therapy (MDT) aims to create dead ends for metastatic pathways. Historically, prostate cancer (PCa) metastases were identified by computed tomography (CT) or bone scan, and androgen deprivation therapy (ADT) was initiated. While highly effective, systemic therapy is toxic. The STAMPEDE and LATITUDE trials [8, 9] had 33% and 48% (ADT alone) and 47% and 63% (combination) ≥ grade 3 toxicity in the respective arms. Prospective studies on positron-emission tomography (PET)-based MDT have shown improvements in progression-free survival (PFS) or ADT-free survival (ADT-FS) with moderate toxicity but had either small cohorts, used dated PET tracers, included different types of metastases (nodal, bone, visceral), treated at higher PSA values, allowed concurrent ADT, and also used conventional radiotherapy (RT) and surgery as MDT. Current prostate-specific membrane antigen (PSMA)-based imaging can detect nodal oligometastases at PSA values of < 0.5 ng/ml [10]. We hypothesized that early and—if necessary and feasible—repetitive stereotactic body radiotherapy (SBRT) based on modern PSMA-PET tracers would improve freedom from palliative treatment (ADT, chemotherapy [ChT], and palliative RT) with low toxicity compared to results of published prospective studies ([11–16]; Table 1).

**Table 1:** Clinical studies in oligorecurrent (OR) prostate cancer (PCa) treated with metastases-directed therapy (MDT)

Year	First author	Journal	Type of study	Number of patients	Number & type of metastases	Distribution of metastases	PSA/testosterone levels	PSA-dt	Handling of systemic therapy	Prior ADT rate	Current ADT rate	Diagnostics (screening and planning)	MDT intervention	SBRT dose & fractionation	Duration of FU	PSA-PFS	Local control	ADT-FS	DPFS	Toxicity‑/quality of life score	Toxicity
2018	Ost	*J Clin Oncol*	Phase II	62	1–3 asymptomatic metastases	Nodal: *n* = 17 (54.8%); non-nodal: 14 (45.2%)	Testosterone < 50 ng/ml excluded; median PSA 3.8 ng/ml (surveillance) and 5.3 ng/ml (MDT)	All, but stratified between ≤ 3 vs. > 3 months	Concurrent ADT or chemo and PSA-influencing drugs within last month excluded	48.4% (surveillance) and 38.7% (MDT)	None	Choline-PET-CT	SBRT or surgery (single node or in case of pelvic involvement bilateral salvage PLND) vs. surveillance	30 Gy in 3 fx	3 years	Median time to PSA progression 10 months	n. s.	13 months (surveillance) and 21 months (MDT)	n. s.	QLQ-C30, PR25	Grade 2 or higher: none
2018	Siva	*Europ Urol*	Prospective interventional clinical trial	33	1–3 bone or lymph node metastases	20 bone only, 12 node only, 1 both	Median PSA 6.4 ng/ml	n. s.	Prior chemo excluded, change in ADT within last 6 weeks excluded	18% (*n* = 6) castration resistant	33% (*n* = 11) on ADT	Screening: bone scan or CT; treatment planning: sodium fluoride positron-emission tomography (PET)/CT scan, 2nd PET at 12 months	SABR	20 Gy single fraction	Follow-up after SABR was every 3 months for 2 years	n. s.	97% at 1 year, 93% at 2 years	22 patients who were castration sensitive and did not have ADT at the time of SABR. In this subgroup the freedom from ADT treatment at 24 months was 48%	58% at 1 year, 38% at 2 years	EORTC QLQ-C30, QLQ-BM22 (bone metastases)	Grade 2: diarrhea (*n* = 1), back pain (*n* = 2), fracture (*n* = 2), myositis (*n* = 1), neuralgia (*n* = 1), any AE (*n* = 5); grade 3: fracture (*n* = 1), any AE (*n* = 1)
2018	Kneebone	*Europ Urol*	Prospective, single-center observational study	57	1–3 metastases	Bone only 18 (31%), lymph node only 37 (65%), bone and lymph node 2 (4%)	Mean 2.12 ng/ml	n. s.	Any systemic therapy excluded	5 had EBRT and ADT, 10 had RP and RT and ADT, 20 had RP ± ADT	Excluded	PSMA_PET CT for screening and planning	SBRT	Nodal: 50 Gy in 5 fx for high-dose volume and 30 Gy in 5 fx for surrounding low-dose volume, later 30 Gy in 3 fx and 24 Gy in 3 fx. Bone: 20 Gy in 1 fx or 24 in 2 fx.	16 months	11 months, 31.9% bDFS at 15 months	100%	n. s.	n. s.	CTC-AE v.4.0	Grade 2: incontinence (*n* = 1). grade 3: none
2020	Pillips	*JAMA Oncol*	Phase II	54	1–3 asymptomatic metastases no larger than 5 cm in the largest axis or 250 cm^3^	n. s.	≥ 0.5 ng/mL but ≤ 50 ng/mL	Stratified by PSA-dt < 6 months vs. 6–14.9 months	No ADT within last 6 months	Not within 6 months of enrollment, 15 in SABR arm	Excluded	Screening: CT, MRI, bone scintigraphy; treatment planning: 18F-DCFPyL-PSMA-PET/CT	SBRT vs. surveillance	19.5–48.0 Gy in 3 to 5 fx	18.8 months	SBRT: not reached, observation: 5.8 months; 89% at 6 months, median PFS not reached in patients with no untreated lesion, 11.8 months in patient with untreated lesions	98.9% at 6 months	n. s.	n. s.	CTC-AE v.4.0	New grade 2 AEs at 90 days: 3% incontinence, 3% esophagitis, 3% dizziness; new grade 2 AEs at 180 days: 3% incontinence, 3% bladder infection
2021	Supiot	*Euro Urol*	Multicenter prospective phase II study	67	1–6 pelvic lymph node metastases; local recurrences after surgery allowed	Only pelvic lymph node metastases	Median PSA at baseline groups A & B (prior RP, prior RP & prostatic bed relapse): 3.9 ng/ml; groups C & D (prior RP & prostatic bed RT, prior RT [EBRT & BT]): 3.6 ng/ml	Group A & B. 4.6 months, group B & C: 5.2 months	No ADT within last 6 months	Groups A & B: 2 (6.4%); groups B & C: 10 (28%)	Excluded	FCH-PET	EBRT	Whole pelvis 1.8/54 Gy, SIB to PLNs 2.2/66 Gy; patients without prior prostatic bed RT: prostatic bed 2/66 Gy, macroscopic prostatic bed relapse: 2/72 Gy	Median follow-up for survivors 49.4 months (IQR: 42.3–53.1 months)	Median BRFS was 25.9 months	n. s.	Median TTST (time to secondary treatment) was 49.8 months (SBRT, ADT, or both; median TTADT [time to ADT]) was 51.9 months	n. s.	CTC-AE v.4.0, QLQ-C30, PR-25	Grade 2 at 2 years: anal/rectal (*n* = 2), bowel (*n* = 1), sexual (*n* = 4), urinary (*n* = 3), other (*n* = 2); grade 3: sexual (*n* = 1), urinary (*n* = 2)
2021	Glicksman	*Europ Urol*	Investigator-initiated, single-institution, open-label, single-arm phase-II trial	37	1–5 metastases (92% lymph node, 82% pelvic lymph nodes)	Lymph node: 34 (92%) (miN1a [pelvic single LN] 15 (41%), miN1b (pelvic multiple LN) 15 (41%), miM1a (non-pelvic LN) 2 (5%), miN1 and miM1a 2 (5%), bone 3 (8%)	PSA values at enrollment 0.4–3.0 ng/mL with normal age-adjusted testosterone levels	Median 16.1 months	Allowed if 1 year had elapsed after last gonadotropin-releasing hormone agonist injection and with recovered testosterone levels	6 (16%) had PORT + ADT	None	Screening: CT abdomen, pelvis, and BS; planning: 18F-DCFPyL PET-MR	SABR or surgery	27–30 Gy in 3 fractions	15.9 months	Median time to PSA progression 17.7 months	n. s.	Median time to ADT was not reached	n. s.	CTCAE v4.0	Grade 2 (*n* = 1), grade 3 (*n* = 1)

*PSA* prostate-specific antigen, *ADT* androgen-deprivation therapy, *SBRT* stereotactic body radiotherapy, *FU* follow-up, *ADT-FS* time to ADT, *PET* positron-emission tomography, *EBRT* external-beam radiotherapy, *PSMA* prostate-specific membrane antigen, *bDFS* biochemical disease-free survival, *PORT* postoperative radiotherapy, *SIB* simultaneous integrated boost, *TTST* time to systemic treatment, *BRFS* biochemical recurrence-free survival

## Methods

This was a retrospective analysis of patients treated with CyberKnife (CK) (Accuray, Inc., Sunnyvale, CA, USA) or Novalis (NV; Brainlab AG, Munich, Germany/Varian Medical Systems, A Siemens Healthineers Company, Palo Alto, CA, USA) for nodal OR PCa after curative first-line therapy at the Department for Radiation Oncology, Charité – University Medicine Berlin, Germany. Patients with predominantly bone OR and concurrent systemic treatment were excluded. All treatments were managed according to Good Clinical Practice and the German Radiation Protection Laws. The research complied with the Declaration of Helsinki. The Charité ethics board approved this study (EA2/110/21). Since data were stored anonymously, the informed consent requirement was waived.

**Fig. 1:**
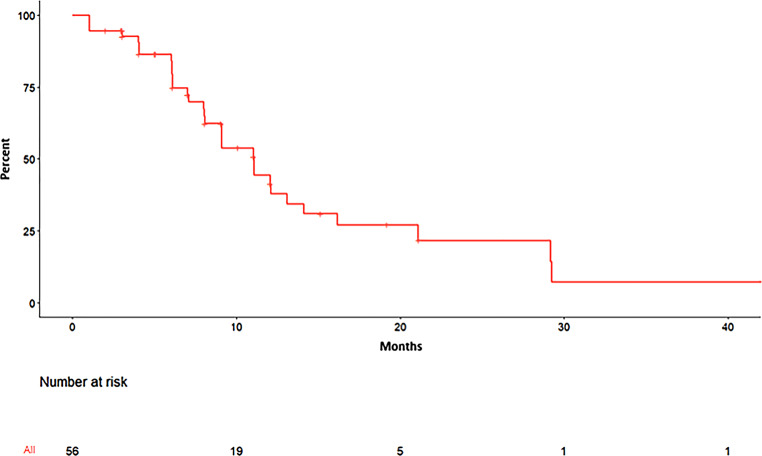
Progression-free survival by prostate-specific antigen (PSA). Any PSA increase ≥ 0.2 ng/ml was considered a PSA progression. Mean (median; range) PFS for the whole cohort (this graph) was 9.5 (7.5; 1–45.3) months. Mean (median; range) PFS for single-course SBRT (*n* = 33) and multiple-course SBRT (*n* = 31) was 9.4 (6.1; 2–45.3) and 9.5 (8; 1–29.2), respectively (*p* = 0.4096; number at risk 56, 8 patients with missing data, 3 patients lost to follow-up)

**Fig. 2:**
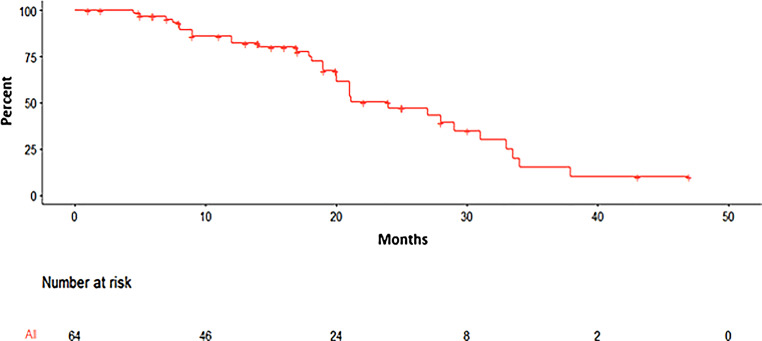
Stereotactic body radiotherapy (SBRT)-free survival (SBRT-FS). Not every increase in PSA automatically triggers new interventions; hence, SBRT-FS better reflects the true freedom from any treatment compared to PFS alone. SBRT-FS was defined as the time between one SBRT series to the next, if applicable. Mean (median; range) PSA in ng/ml for the whole cohort (*n* = 64, this graph), single-course SBRT (*n* = 33), multiple-course SBRT (*n* = 31) was 17.3 (17.0; 0–47), 15.4 (14.0; 0–47), and 19.4 (19.5, 4.5–37.9), respectively (*p* = 0.06; number at risk 64, 3 patients lost to follow-up)

**Fig. 3:**
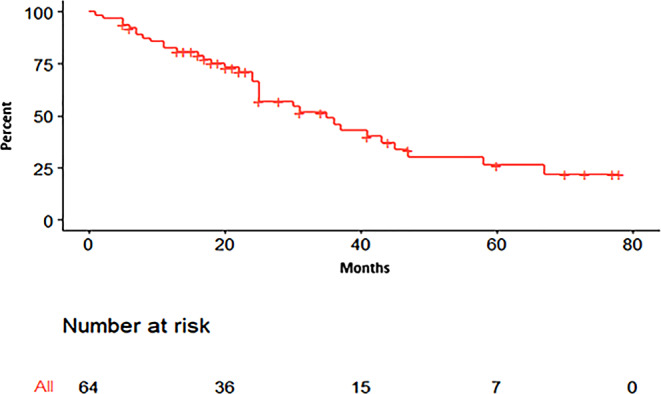
Androgen deprivation therapy (ADT)-free survival (ADT-FS). The goal of metastases-directed treatment (MDT), in this case SBRT, was to postpone palliative treatment. ADT-FS was mainly driven by freedom from actual ADT, but we also included freedom from chemotherapy and freedom from palliative EBRT into ADT-FS. Mean (median; range) ADT-FS for the whole cohort (this graph, *n* = 64); the patients experiencing treatment escalation by means of ADT, chemo, or palliative EBRT (*n* = 34); and the patients reaching the end of follow-up without treatment escalation (*n* = 30) was 27.5 (22.1, 1–78.1), 23.4 (23.0, 1.0–67.0), and 32.1 (22.1, 5.0–78.1), respectively (*p* = 0.18; median ADT-FS for patients with multiple-course SBRT [*n* = 31] was 35.2 months; number at risk 64, 3 patients lost to follow-up)

Prostate-specific antigen (PSA) recurrence (= biochemical recurrence [BR]; at least three consecutive rising counts) after either surgery (radical prostatectomy [RPE]) ± RT (prostatic bed ± pelvic lymphatics) or definitive RT [17] triggered PSMA-PET imaging with subsequent identification of nodal OR. The PET/CTs were either obtained externally or through the Charité Department for Nuclear Medicine. The methodology has been described previously [18]. After definitive radiotherapy, we deliberately did not wait until the Phoenix criteria had been met because we wanted to identify patients with early oligorecurrences and analyze how they would benefit in terms of our endpoints. The recurrent node(s) constituted the gross target volume (GTV) with a 1–5 mm isometrically enlarged PTV (planning target volume; location dependent). The dose schedule for Cyberknife (CK) plans was 1 × 19–21 Gy or 3 × 8 Gy (to the surrounding 70% isodose line) and 3 × 10 Gy for Novalis (NV) plans (prescribed to 100%, and the 80% isodose line covered the PTV). Techniques and constraints were according to published data [19–22]. Treatment intervals were 24–48 h [23] depending on the proximity of organs at risk (OAR). Toxicity data were gathered from electronic and physical files, 64 patients (96%) could also be contacted by phone. Toxicity was scored using the Common Terminology Criteria of Adverse Events version 5 (CTCAE v5). Endpoints of this study were bPFS, SBRT-FS, ADT-FS, and toxicity. They were calculated from the start of SBRT. PFS was defined as any PSA increase from nadir > 0.2 ng/ml or death (Fig. 1). SBRT-free survival (SBRT-FS) was defined as the time between the first and next course of SBRT in the case of re-SBRT for further nodal recurrences or death (Fig. 2). ADT-free survival (ADT-FS) was defined as the time between the first course of SBRT and the initiation of any systemic or palliative local treatment (ADT, ChT *n* = 1, palliative RT *n* = 1) or death (Fig. 3). After SBRT, there was no predefined PSA value triggering re-staging, re-SBRT, or initiation of palliative treatment (ADT, ChT, or palliative RT), hence the data reflect the treating physicians’ discretion. Repeat PET scans were not obtained routinely, but rather only in the case of PSA recurrence after SBRT. For survival analyses, hazard ratios, and PSA distribution analysis, we used the Kaplan–Meier method, the Cox proportional hazard model, and Shapiro–Wilk and Kruskal–Wallis tests. *P*-values < 0.05 were deemed significant. We refer to individual patients, individual metastases, and/or treatment series to avoid conflicting information (e.g., different PET tracers per series).

## Results

Between 11.15.2013 and 01.15.2021, 67 patients and 248 metastases (211 nodal = 85.1%) were treated in 122 series (Table 2). Median follow-up was 22 months (range 1–78). All patients were ECOG 0–1. Median PSA at the time of SBRT was 2.175 ng/ml (range 0.01–43.8). Median PET-to-SBRT time was 2.0 months (0.9–5.1). Median number of metastases per patient was 1.9 (range 1–13). The CyberKnife/Novalis ratio was 82.6%/17.4%. SBRT was either single dose (40.8%) or fractionated (57.8%). 5.5% of patients needed fiducials. Median (range) GTV and PTV volumes were 1.1 (0.05–30.8) and 3.9 (0.2–67.2) ml, respectively. Median estimated delivery time for patients treated on the CyberKnife was 49.5 min (range 22–87). Estimated treatment times for the Novalis plans are not given but range around 10 min. The median time to nadir was 4.0 months (range 1.0–20.0). Median PSA nadir after SBRT was 0.585 ng/ml (range 0–26.8). In patients with repeat PET scans after SBRT, we found 10 local recurrences, yielding a local control rate of 81.8% in this subgroup. Patients with re-SBRT had a median ADT-FS of 35.0 (range 7.0–78.1) months (Fig. 3). Median ADT-FS was significantly affected by the number of metastases (≤ 3 vs. ≥ 4: 22.1 vs. 7.0 months).

**Table 2:** Patients’ past and current treatment characteristics

*n* = 67 patients, 122 series	
Age (years)	Mean 62.6 (median 61.9, range 42.4–78.5)
< 60 years	29 (43.3%)
> 60	36 (56.7%)
***First-line therapy***	
*RPE*	*61 (91%)*
*EBRT*	*6(7.5%)*
*Other*	*1(1.5%)*
***Initial PSA at first-line therapy (ng/ml)***	*Mean 17.5 (median 9.5, range 1.9–146)*
***Biopsy Gleason score***	
*6*	*2 (4.1%)*
*7a*	*4 (8.2%)*
*7b*	*9 (18.4%)*
*8*	*19 (38.8%)*
*9*	*13 (26.5%)*
*10*	*2 (4.1%)*
*Missing*	*18*
***Post-RPE Gleason-score***	
*6*	*2 (3.5%)*
*7a*	*11 (19.3%)*
*7b*	*15 (26.3%)*
*8*	*19 (33.3%)*
*9*	*19 (33.3%)*
*10*	*1 (1.8%)*
*Missing*	*10*
***Post-RPE T-stage***	
*T2b*	*2 (3.4%)*
*T2c*	*15 (25.9%)*
*T3a*	*8 (13.8%)*
*T3b*	*29 (50%)*
*T4*	*1 (1.7%)*
*Missing*	*3*
***Post-RPE N-stage***	
*N0*	*46 (79.3%)*
*N1*	*12 (20.7%)*
*Missing*	*4*
***M-stage***	
*M0*	*21 (84%)*
*Mx*	*4 (16%)*
*M1*	*0*
*Missing*	*42*
***Resection status***	
*R0*	*32 (55.2%)*
*R1*	*25 (43.1%)*
*R2*	*1 (1.7%)*
*Missing*	*9*
***Prior EBRT to prostate/prostatic bed***	
*No*	*21 (31.3%)*
*Yes*	*46 (68.7%)*
*Missing*	*0*
***Prior EBRT pelvic lymphatics***	
*No*	*57 (85.1%)*
*Yes*	*10 (14.9%)*
*Missing*	*0*
***Prior ADT (patients n*** ***=*** ***67)***	
*No*	*45 (67.2%)*
*Yes*	*22 (32.8%)*
*Missing*	*0*
***Prior ADT (series n*** ***=*** ***122)***	
*No*	*95 (77.9%)*
*Yes*	*27 (22.1%)*
*Missing*	*0*
***Prior ChT***	
*No*	*67 (100%)*
*Yes*	*0*
*Missing*	*0*
**PET tracer (per series)**	
F18	20 (16.4%)
Ga68	100 (82%)
**Type of metastasis (per series)**	
Nodal	99 (81.2%)
Bone	12 (9.8%)
Both	11 (9.0%)
**Nodal metastases**	
Number/ratio	211 (85.1% of 248)
Range	1–13
Median	5
**Nodal metastases pelvis**	
Number/ratio	153 (72.5%)
Range	1–5
**Nodal metastases paraaortic region**	
Number/ratio	57 (27%)
Range	1–12
**Nodal metastases thorax**	
Number/ratio	1 (0.5%)
Range	1
**Bone metastases**	
Number/ratio	37 (14.9%)
Range	1–7
**Bone metastases pelvis/sacral spine**	
Number/ratio	10 (27%)
Range	1–2
**Bone metastases lumbar spine**	
Number/ratio	5 (13.5%)
Range	1
**Bone metastases thoracic spine/rips**	
Number/ratio	20 (54.2%)
Range	1–6
**Further OR after SBRT (per series)**	
No	57 (46.7%)
Yes, local failure	3 (2.5%)
Yes, new site	55 (45.1%)
Yes, local failure and new site	7 (5.7%)
Local control	112 (91.8% of 122)
**Prior treatment for OR PCa (per patient)**	
No	55 (82.1%)
Yes	12 (17.9%)
**SBRT courses for OR PCa**	
Median (range)	1 (1–7)
1	67
2	32
3	12
4	4
5	3
6	2
7	2
**Time from initial diagnosis to OR (months)**	
Median (range)	58 (9–258)
**Time from first to second OR (months; *n*** **=** **32)**	
Median (range)	20 (4–49)
**Time from second to third OR (months; *n*** **=** **12)**	
Median (range)	13 (1–33)
**Pre-SBRT PSA**	
Median (range)	2.175 (0.01–43.8)
**Post-SBRT PSA nadir**	
Median (range)	0.585 (0–26.8)

The italic area depicts all previous staging and treatment information before diagnosis of oligo-recurrent PCa

The nonitalic area highlights the data at the time of treatment for oligorecurrent PCa

*RPE* radical prostatectomy, *EBRT* external-beam radiotherapy, *ADT* androgen deprivation therapy, *ChT* chemotherapy, *PET* positron-emission tomography, *SBRT* stereotactic body radiotherapy, *OR* oligorecurrence, *PCa* prostate cancer, *PSA* prostate-specific antigen

PFS, SBRT-FS, and ADT-FS were not affected by age (≤ 60 vs. > 60 years), initial PSA (< 10 ng/ml vs. 10–20 ng/ml vs. > 20 ng/ml), first-line treatment (RPE ± postoperative RT vs. definitive RT), percentage of positive scores in the initial biopsy (< 50% vs. ≥ 50%), initial Gleason scores (≤ 6, 7a, 7b, ≥ 8), initial resection status (R0 vs. R1/2), initial PSA persistence after RPE (yes vs. no), PET-to-SBRT time (≤ 3, ≤ 6, > 6 months), type of metastases (nodal vs. bone vs. both), location of metastases (paraaortic vs. thoracic), time to OR (≤ 58 vs. > 58 months after initial diagnosis), or pre-SBRT PSA (≤ 2 vs. > 2 ng/ml) [13].

The overall toxicity rate was 69% (*n* = 46), consisting of grade 1 nausea (*n* = 17), fatigue (*n* = 12), pain (*n* = 8), diarrhea (*n* = 7), and bloating (*n* = 2). There was no ≥ grade 2 toxicity.

## Discussion

Nodal OR PCa can be detected at PSA levels ≤ 0.5 ng/ml. Early focal SBRT to small lymph nodes based on modern-tracer PET is an efficient outpatient procedure with ablative doses and without significant toxicity. We aimed to further improve ADT-FS compared to results from already published prospective studies by setting a tight definition of bPFS, obtaining early re-PET scans, and offering re-SBRT if applicable. We included 67 patients (248 metastases), which is, except for the study by Supiot et al., more than in any published prospective series. There are retrospective studies on mixed-type metastases with more patients [24]. All our patients received ^68^Ga- [25] or ^18^F- [26] PSMA-PET/CT at a median PSA level of 2.175 ng/ml. Early PET tracers like choline have been shown to be inferior to PSMA-PET/CT (especially at low PSA values) [27–32]. Some groups treated at higher PSA values (Ost et al. median 5.3 ng/ml in the MDT arm; Siva et al. median 6.4 ng/ml; Phillips et al. up to 50 ng/ml). We focused on patients with early NOR (range per patient 1–12). Ost et al. and Kneebone et al. had 45.2% non-nodal and 31% bone-only metastases. 32.8% of our patients had ADT as part of their first-line treatment. Siva et al. had 33% of patients on concurrent ADT. Ost et al. allowed ChT and ADT except for a month prior to treatment. Testosterone recovery after ADT exceeds 1 month; hence, persistent effects can be assumed [33, 34]. Phillips, Supiot, and Glicksman et al. had a mandatory interval of 6–12 months to prior ADT. Our patients had 1–3-fraction SBRT (CK [82.6%] or NV [17.4%]). Ost et al. and Glicksmann et al. included SBRT or surgery (pelvic lymphadenectomy, i.e., elective treatment). Supiot et al. administered whole-pelvis ± prostatic bed RT with a boost to involved nodes [35, 36]; they reported 13.4% and 14.9% grade 2 acute GU (genitourinary) and GI (gastrointestinal), respectively, and 4.4% grade 3 acute GU toxicity [37]. SBRT dose and fractionation varied between 20 Gy single-dose treatment [12] to fractionated approaches of 19.5–50 Gy in up to 5 fractions [13] We offered single-dose or 3‑fraction treatment in 40.8%/57.8%. Local control in published series varied between 98.9% at 6 months [14] (progression in imaging defined by RECIST or trial radiologist), 97% at 1 year, and 93% at 2 years [12] (RECIST progression in imaging and PET confirmation) to 100% during an FU of 16 months in Kneebone et al. (any CT progression). PET-assessed local control in our subgroup of patients with repeat PET scans was 81.8%. Our local control rate is negatively biased by the fact that we did not obtain re-PET/CTs for all patients. Hoelscher et al. [38] graded the types of failures according to the class of progression schema proposed by Deek et al. [39]. Our median PFS for the whole cohort, single-course SBRT, and multiple-course SBRT was similar to that in Ost et al. and Kneebone et al., with 10 and 11 months, respectively. However, in the GETUG trial, PFS was significantly longer, with a median of 25.9 months, but with a higher rate of toxicity, longer treatment time, and exclusion of metastases outside the pelvis. Our median (range) ADT-FS was 23 (1–67) months. Approximately 20% of patients reached the end of FU without treatment escalation (median 22.1, range 5–78 months). In the STOMP trial, ADT-FS was 21 months [11]. Siva et al. describe a subgroup of castration-sensitive patients with a freedom-from-ADT rate at 2 years of 48% [12]. In Glicksmann et al., the median time to ADT was not reached at a median FU of 15.9 months. In the GETUG trial, the median time to ADT initiation was 51.9 months (previously mentioned caveats). The time to re-SBRT was not systematically given in published studies. Patients with multiple SBRT in our cohort had a median ADT-FS of 35.0 months. Comparable to the STOMP trial, we saw no grade 2 or higher AE. Siva et al. describe 7 patients with grade 2 and 2 patients with grade 3 toxicity. Because of the large elective volumes treated, Supiot had increased grade 2 and 3 acute toxicity [37]. At 2 years they still had 12/67 patients with grade 2 and 3/67 patients with grade 3 toxicity. Glicksmann et al. report on 1 patient each with grade 2 and 3 events [16].

Outside of prospective trials, Kwon et al. report on 25 patients (with 10 already castration resistant) showing an ADT-FS/ADT-escalation-FS of 23.1 months using novel-tracer PET-based SBRT to PCa oligometastases [40]. Kipper et al. performed PSMA radio-guided surgery in 364 patients with OR PCa yielding a median PFS of 7.8 months but a median therapy-free survival of 35.5 months, which dropped to 19.7 months in patients with ≥ 2 metastases. Higher-grade AE were < 7% [41].

## Strengths and limitations

The strengths of this work are the large number of metastases, the uniform treatment regimens, and very good ADT-FS in patients with multiple SBRTs. The study should pave the way for further MDT optimization.

There are critical issues and shortcomings concerning this analysis. The main weakness is the lack of a control arm. Also, the retrospective nature of this work is susceptible to fault. We included patients with common iliac nodal recurrences as M1a oligo, although prognostically, they show similar outcomes to pelvic N1 patients and might thus have been amenable to extended-field curative pelvic ADT-RT [42]; hence, selection bias might have influenced the results. Since there was no predefined PSA cut-off value triggering re-staging, re-SBRT, or the initiation of palliative treatment (ADT/ChT/palliative EBRT), the data might on one hand be skewed by the individual treating physician’s discretion but on the other reflect clinical practice.

## Conclusion

Early initiation of ADT is still not unambiguously recommended [43–45] due to the risk of overtreatment and wastage of an effective but also toxic and finite approach. MDT in oligorecurrent PCa to prevent disease progression is a rapidly growing approach [46, 47]. SBRT is ablative, comfortable, and often free of relevant toxicity. These results should, however, be confirmed in prospective studies. Future analyses should focus on optimization of PET tracers to further reduce the blind window at very low PSA values and on combination approaches with elective volume irradiation and/or temporary ADT.

## Data Availability

All data supporting the results reported in this article are available on a secure data server owned by the Charité—Universitaetsmedizin Berlin, Germany. The datasets and analyses of all data in this manuscript are available from the corresponding author upon reasonable request.
